# “If It Ever Really Hurts, I Try Not to Let Them Know:” The Use of Concealment as a Coping Strategy Among Adolescents With Chronic Pain

**DOI:** 10.3389/fpsyg.2021.666275

**Published:** 2021-06-03

**Authors:** Emily O. Wakefield, Rebecca M. Puhl, Mark D. Litt, William T. Zempsky

**Affiliations:** ^1^Division of Pain and Palliative Medicine, Connecticut Children’s, Hartford, CT, United States; ^2^Department of Pediatrics, School of Medicine, University of Connecticut, Farmington, CT, United States; ^3^Rudd Center for Food Policy and Obesity, University of Connecticut, Hartford, CT, United States; ^4^Department of Human Development and Family Sciences, University of Connecticut, Storrs, CT, United States; ^5^Division of Behavioral Science and Community Health, University of Connecticut Health Center, Farmington, CT, United States

**Keywords:** adolescents, chronic pain, concealment, coping, stigma

## Abstract

**Objective:**

Despite considerable evidence of chronic pain in adolescents, and its adverse consequences for their health and well-being, less is known about pain-related stigma that these youth face, such as pain disbelief by others. Adolescents with chronic pain may conceal their symptoms as a coping strategy to avoid pain-related stigma, contributing to further social isolation and disruptions in medical treatment. In the current study, we used focus group methodology to examine adolescent motivations for using concealment and the possible benefits and harmful consequences of this form of coping.

**Materials and Methods:**

Five focus groups of 3–5 adolescents (ages 12–17) with chronic pain conditions (*N* = 18) were conducted as a part of a larger study to evaluate the impact of, and reaction to, pain-related stigma. Patients were recruited from an outpatient pediatric pain management clinic. Transcripts of focus group sessions were analyzed using directed content analysis for the main study, yielding anticipatory stigma and concealment categories. These categories were then explored using inductive content analysis for the current study.

**Results:**

Adolescents described engaging in concealment of their pain symptoms. Our analysis revealed three social motivations for concealment: (1) avoidance of judgment; (2) avoidance of being a social burden; and (3) desire to be treated normally, and two harmful consequences of concealment: (1) social isolation and (2) cognitive burden.

**Conclusion:**

Disbelief of pain symptoms may exacerbate the social isolation and disease-related burden in this population. Clinical implications of concealing pain symptoms are discussed, and points of intervention are proposed.

## Introduction

Chronic pain in youth has been on the rise over the past 25 years, with a prevalence of approximately one in every 4–5 adolescents (King et al., 2011). Despite the prevalence of pediatric chronic pain, diagnostic ambiguity commonly exists for youth with chronic pain complaints (Betsch et al., 2017; Neville et al., 2019; Tanna et al., 2020). Youth with chronic pain, and their families, experience this diagnostic uncertainty in the context of repeated negative medical findings (Neville et al., 2019), alongside providers who may not feel confident in their management of pediatric chronic pain (Neville et al., 2020). Many parents have also indicated feeling that their child’s pain symptoms are often dismissed by pediatric providers (Igler et al., 2020). The invisibility of chronic pain symptoms has led to adolescents with chronic pain experiencing pain-related stigma, specifically in the form of others indicating that they do not believe their pain is “real,” and/or believing that the person is making up their symptoms (Wakefield et al., 2018).

Stigma occurs when an individual has a specific attribute that is socially undesirable (Goffman, 1963) that can lead to discrimination by others. There are different types of stigma including felt, anticipatory, and internalized (Major et al., 2018). Felt stigma occurs when the stigmatized individual perceives that they are being treated unfairly based on their stigmatizing attribute. Within the context of pediatric chronic pain, felt stigma is experienced when youth with chronic pain perceive that others are negatively judging them or not believing their pain symptoms. Anticipatory stigma refers to negative judgment or treatment that individuals with stigmatized identities expect to experience if others become aware of their stigmatized condition.

Internalized stigma (also called self-stigma) occurs when the stigmatized individual engages in self-blame and applies negative stereotypes to themselves. The anticipation of pain-related stigma may motivate adolescents with chronic pain to conceal their condition from others. While the literature is limited, all of these forms of stigma have been reported by youth with chronic pain conditions (Wakefield et al., 2018; Laird et al., 2020).

When faced with possible negative judgment or treatment by others, individuals who experience stigma may attempt to hide their stigmatizing identity, especially if this identity can be concealed (Quinn and Earnshaw, 2013), such as chronic pain. This coping strategy is known as “concealment,” and research on its impact on health outcomes has been mixed (Camacho et al., 2020). Whereas the lack of disclosure of illness may allow individuals with concealable stigma identities to avoid potential negative treatment by others (Quinn and Chaudoir, 2009; Barned et al., 2016; Benson et al., 2017; Galano et al., 2017), some evidence suggests that concealment leads to poorer physical and psychological well-being (Frable et al., 1998; Quinn et al., 2017; Laird et al., 2020), possibly due to the consequences of internalizing distress. Concealment also prevents access to social support, which is a protective factor in adapting to chronic pain (Ross et al., 2018).

For youth with chronic pain conditions, the empirical evidence on disclosure or concealment of pain symptoms is limited. Some research has linked concealment of chronic pain to negative physical health outcomes (Laird et al., 2020), worse psychological well-being, and lower pain tolerance (Uysal and Lu, 2011; Laird et al., 2020). For example, Uysal and Lu (2011) observed that healthy young adults who demonstrated greater levels of concealment exhibited lower pain tolerance in response to a cold pressor task, indicating that concealment may lead to heightened reactivity to pain stimuli. The inhibition or internalization of emotions that occurs when one engages in concealment can also have a physiological cost, as demonstrated in individuals with trauma (Pennebaker and Beall, 1986). Collectively, these initial findings demonstrate the potential harm that may result from concealing chronic pain conditions to others, but less is known about the experience of pain-related stigma and motivations among adolescents with chronic pain to use concealment as a coping strategy.

To begin to address these gaps in this emerging field of study, the present study aimed to examine the nature of concealment as a coping strategy used by adolescents with primary chronic pain, using focus group methodology. Focus group methodology was used in this case to develop themes that described the experiences and coping behavior of the adolescents with chronic pain without the constraints on responding imposed by established questionnaires or interviews. Thus, focus groups allow the emergence of responses that might not become clear using other means of data collection (Morgan and Krueger, 1993). Due to the benefits of peer connections in the context of social difficulties, and the ability to understand similarities and differences in experiences, focus group methodology was justified. The use of focus groups also provides in-depth descriptions of motivations for concealment as well as observed benefits and harmful consequences. Due to the qualitative nature of the study, there were no specific hypotheses developed *a priori*; however, it was expected that the adolescents in this study would discuss the use of concealment given the previous literature on concealment in the context of health-related stigma (Quinn, 2018).

## Materials and Methods

### Participants

The current study was a secondary analysis of data collected in a larger study evaluating pain-related stigma among adolescents with chronic pain, which was approved by the Institutional Review Board at Connecticut Children’s. The recruitment and procedures of the current study and larger study are the same. Patients with chronic pain receiving treatment in a tertiary multidisciplinary pain management clinic at a midsize children’s hospital were screened for eligibility and approached by research personnel either in person during their outpatient clinic visit or via phone shortly after (within 2 weeks) their appointment. Inclusion criteria for the study was adolescent patients, ages 12–17 years, with a documented diagnosis of chronic pain. Exclusion criteria included the presence of a comorbid chronic medical condition, such as juvenile rheumatoid arthritis or diabetes, in order to reduce the potential stigma bias attributed to non-chronic or identifiable pain conditions. Additionally, adolescents who were not fluent in English were excluded because the focus groups were conducted in English. Data collection occurred from December 2017 to October 2019.

Of the 25 adolescents approached for the study, 18 agreed to enroll in the study. The seven patients who did not enroll were interested, but had scheduling conflicts with the focus group dates. No patients were excluded based on study eligibility criteria. Of those who participated, 88.89% were female (5.56% male, 5.56% gender fluid) and the mean age was 15.33 years (SD = 1.28, range = 12–17). Regarding pain conditions, 13 of the 18 had pain in more than one location (72.22%). Additional demographic information is presented in Table 1.

**TABLE 1:** Demographic information for adolescent participants (*N* = 18).

Demographic characteristics	Number of participants	Percentage of sample (%)
**Gender**		
Female	16	88.89
Male	1	5.56
Gender fluid	1	5.56
**Race/ethnicity**		
White, Non-Hispanic	11	66.11
White, Hispanic	3	16.67
Multi-race, Non-Hispanic	2	11.11
Black, Hispanic	1	5.56
Other, Hispanic	1	5.56
**Pain diagnoses**		
Amplified musculoskeletal	11	66.11
Pain syndrome		
Abdominal pain	6	33.33
Pain amplification syndrome	5	27.78
Back pain	4	22.22
Chest pain	2	11.11
Irritable bowel syndrome	2	11.11
Complex regional pain syndrome	1	5.56
Fibromyalgia	1	5.56
Neck pain	1	5.56
Migraine	1	5.56

### Procedures

Parental/legal guardian written informed consent and adolescent assent were obtained prior to the meeting of the focus group. The groups were conducted in person by a child clinical psychologist trained in qualitative interviewing and experience working with adolescents with chronic pain. The focus groups were conducted using a semi-structured format. Group rules were discussed prior to the start of the focus group. Rules included show respect for different experiences, maintain confidentiality of content discussed following the group, and participants need only answer questions to the extent to which they are comfortable.

A question route was developed prior to the focus groups (see Table 2) as suggested by Krueger and Casey (2015) to provide a sequential question structure that introduced the concept of pain-related stigma gradually. The key questions targeted five primary areas: (1) participants’ experiences with initial diagnosis and treatment by medical providers; (2) participants’ perceived reactions and level of support from teachers/school staff, family members, and peers; (3) participants’ perceptions of exclusion or being treated unfairly; (4) participants’ feelings of being ashamed because of pain; and (5) participants’ perceptions of being teased or negatively judged because of pain. After participants responded to each question, the interviewer probed for additional information to gain more insight into their experiences.

**TABLE 2:** Focus group question sequence.

Question type	Focus group question
Introductory question	Let us go around the room and have everyone introduce themselves by saying what school you go to, grade you are in, and, if you feel comfortable, where you experience pain and how long you have had pain?
Transition question	Has there been challenges you have experienced related to your pain? Tell me more about them?
Key question	Tell me about how your chronic pain was diagnosed. What was your experience of how doctors initially reacted to your pain?
Key question	How have teachers and school nurses reacted to your pain? In what ways have they been supportive? In what ways have they not been supportive?
Key question	Tell me about how other students and friends reacted to your pain? In what ways have they been supportive? In what ways have they not been supportive?
Key question	How do your parents and/or other members of your family support or do not support your pain condition?
Key question	Tell me about any time you have felt excluded or treated unfairly by others because of pain?
Key question	Tell me about any time you have been made to feel ashamed of your pain?
Key question	Tell me about any time you have been teased or judged negatively because of your pain?
Ending question	Of all the topics we have discussed today, which of them would you say are most important to you?
Ending question	If you were to ask other teens about these experiences, what type of questions would you ask? How would you ask them?

To encourage feedback from all participants, the focus group facilitator invited any adolescent who had not shared on a topic area, if they felt comfortable, to include their experiences before moving on to another question. All participants shared their experiences in each topic area. A total of five focus groups were conducted and each focus group consisted of 3–4 participants. The duration of focus groups ranged from approximately 36–106 min.

Focus groups were conducted until no additional stigma information was presented by participants and identified during coding for the larger study. The focus groups were recorded. The recordings were transcribed and the transcriptions were reviewed for accuracy. The transcripts were then reviewed by two independent coders using directed content analysis (Hsieh and Shannon, 2005) who obtained 90.34% agreement (552 code agreements out of 611 total codes). The 59 discordant codes were discussed between the coders to determine consensus.

Stigma theory was used *a priori* to develop a stigma-based codebook, which included anticipatory stigma (defined as the perception of negative future reactions to their chronic pain by others) and concealment, defined as intentionally hiding or not disclosing chronic pain symptoms to others. Responses that included these two categories were extracted from the data for a more focused analysis for the current study using inductive content analysis by one coder. This coder engaged in repeated reading and rereading of the transcripts to develop codes that were organized into categories that reflected emerging themes for the current study. The transcripts were revisited on a regular basis to confirm that the codes and themes were reflected in the data (Hsieh and Shannon, 2005). The research literature on concealment in youth with chronic health conditions was also reviewed to provide additional validation of these categories. The coders for the larger and current study were trained and received supervision in qualitative research analyses.

## Results

The primary study analyses indicated that pain-related stigma experiences were reported by all participants and experienced across diverse social situations (i.e., medical providers, school personnel, family members, and peers). Sources of pain-related stigma included disease invisibility and diagnostic uncertainty. In response to these pain-related experiences, anticipatory stigma and concealment of pain symptoms emerged as consequences of perceived pain-related stigma from others. Anticipatory stigma was a reason for participants to conceal their symptoms. In total, 46 responses were included in the analysis of the current study. All participants except one described the use of concealment. The participant who did not use concealment was the only male in our study and he shared an indifference about whether others knew of his chronic pain status.

Inductive content analyses yielded two main themes of responses related to concealment: (1) social motivations to conceal pain, and (2) harmful effects of concealment. Within social motivations to conceal pain, three categories emerged: (1) avoidance of judgment; (2) avoidance of being a social burden; and (3) desire to be treated normally. Harmful consequences of concealment had two categories: (1) social isolation and (2) cognitive burden. These categories are further described below.

### Social Motivations to Conceal Pain

#### Avoidance of Judgment

Participants discussed several ways that concealment of their pain symptoms had social benefits. The most commonly described social motivation for concealment of pain symptoms was avoidance of judgment from others. In reaction to previous experiences of negative interactions from others, most participants in all the focus groups shared that they hid their pain to avoid negative judgment. The majority expressed using concealment as a coping strategy to avoid judgments with school staff. For example, one 15-year-old female shared, “I wouldn’t tell [my teachers] I’m having a whole bunch of pain…I would get on the bad side of some teachers.” Many participants reported that they had experienced negative interactions with teachers and other school personnel when attempting to engage in school accommodations or services for their pain symptoms. They described teachers or school nurses being “frustrated” with them or questioning the need for the accommodation and, as a result, the adolescent engaged in concealment to avoid these interactions. For example, the same 15-year-old stated, “I told this one teacher at the beginning…he like got really pissed at me, really fast, so I just told him and he again, he didn’t really care or understand. So I just pretty much gave up with that.”

A few participants in all of the focus groups described concealment as a way to avoid judgments from family members. One female participant (age 15 years) shared

“It was more like I was judged by pretty much everyone, even my family and my siblings. My grandmother thought I was just faking it, and pretty much everyone thought that I’m doing it for attention, that it’s not real. It’s all in my head. More like, I was more like, I felt like I learned to stop telling everyone, more like I kept it inside.”

A few participants also described the experience of others believing that they were fabricating their pain symptoms within their own families. Concealment of pain symptoms was described as a way to avoid this judgment.

Specifically regarding peers and friendships, a few participants described using concealment to avoid judgment from peers, but more participants described concealment in the context of avoiding discomfort experienced in reaction to the lack of understanding or empathy. An example of this experience was described by a 15-year-old female participant:

“I’ve tried not to tell most of my friends, especially since they don’t understand what it is. This year, actually I moved, I have a 504 [school accommodations plan] where I can like sit, like so I can just get out of the classroom, just in case I have to take a walk or ask for a drink of water, and I’ll be out for probably like half an hour, 20 min, and this kid didn’t understand so I told him to look it up. He didn’t understand, so pretty much, like I don’t tell anyone, only one person probably knows about it.”

A few participants in each of the focus groups discussed concealment in the context of medical provider interactions. For example, due to the perception that medical providers would dismiss her pain symptoms, a 15-year-old female participant stated, “They kind of look at you. And you look fine. You look like you’re just sitting there, but sometimes you just don’t really want to show them how you feel I guess. And they rely on what they see; not really how you are feeling I guess.”

#### Avoidance of Social Burden

Another social motivation to conceal their symptoms was the perception that disclosing their pain symptoms to others would be too much of a burden on others. Social burden was described as both feeling like their pain condition would be difficult to hear and that, over time, others would stop supporting them. All participants of one focus group and a few in two other focus groups described this social burden in the context of family members and friendships. As a result, some participants concealed their pain symptoms to avoid feeling like a hardship on others. For example, one 16-year-old female participant stated:

“I’m probably ashamed to tell anyone if I have like a new pain because I feel like I’ve already had so much pain that it’s my problem and no one else should have to deal with it…it seems like every time I tell someone, it just adds something else they have to deal with, with everything else they have.”

In addition to feeling like talking about their pain symptoms would be too much for others to manage initially, participants also described the perception that others might stop supporting them over time. The anticipation of reduced support from others motivated participants to conceal their pain. For instance, as a different 16-year-old female participant expressed, “you have these things going on and you can’t complain about every second of every day because otherwise everyone gets pissed off at you.”

#### Desire to Be Treated Normally

The third social motivation to conceal pain involved hiding pain symptoms in an attempt to be socially accepted and seen as “normal.” This social motivation was described in by some participants in most of the focus groups with head nodding by all participants when it was described. For example, a 15-year-old female participant also stated, “you don’t want to be treated like, kind of like, not like you’re a patient, but you want to be treated as if you’re like just regular.” Regarding services in school, a different 15-year-old female participant stated, “when you have a 504 plan [school accommodations], sometimes you kind of forget about what you have, or you try to ignore it, because you want to be as normal as possible and fit in with everybody, so you don’t want to bring it up.” Participants described the desire to feel “normal” mostly in the school setting due to the context of their peers.

### Harmful Consequences of Concealment

Participants also discussed ways in which concealment was harmful to them in the context of their pain symptoms. Two subcategories emerged regarding negative aspects of concealment. First, participants shared that concealment interfered with their ability to either develop friendships or receive support from others. Second, participants reported that it was cognitively exhausting to constantly keep their symptoms to themselves.

#### Social Isolation

Participants described ways in which concealment led to further social isolation from others. Social isolation was described by all participants generally, but a few participants in most of the focus groups described it in the context of their use of concealment. The lack of understanding of the stress of their symptoms contributed to loneliness and social isolation from others. A gender fluid 14-year-old adolescent (she/her preferred pronoun) shared,

“In my school I don’t have like any friends and like if I did, they probably wouldn’t like if they probably want do a lot of stuff but like I can’t like I can’t go to the movies all the time because I can’t walk around…like then I would feel upset because like every day I come home and cry my eyes out because I have no friends.”

This participant experienced significant social isolation that was complicated by the perception that peers would not want to be this persons’ friend due to her pain symptoms, which was very distressing to her. This experience was shared following another female participant (age 12 years) who discussed a “disconnection” due to her inability to describe her pain to her friends. The majority of participants also shared the challenges with social isolation in either not having friendship or experiencing distance from current friendships due to the lack of understanding regarding their chronic pain.

#### Cognitive Burden

In order to successfully conceal pain symptoms, adolescents with primary chronic pain need to be vigilant to cues that may reveal their pain symptoms and attempt to hide those cues. This process creates extra cognitive effort for individuals who attempt to conceal their pain, which has been noted in the coping literature (e.g., Wegner and Lane, 1995). Cognitive burden was described in some of the participants in all of the focus groups. As an example, one 15-year-old female participant shared, “Like you get good at covering for pain or just like not showing it …I think that’s why I’m tired all the time because I’m pushing myself to like cover it up I guess.” Another female participant (age 16 years) similarly shared, “But you can’t hide like it all the time because sometimes it gets to be too much, so it’s sort of regulating that bit.” These examples demonstrate that concealment can contribute to a cognitive burden for adolescents with primary chronic pain due to the cognitive effort that this concealment requires.

## Discussion

Our findings in these focus groups suggest that concealment is used as a coping strategy in the context of pain-related stigma in our sample of adolescents with chronic pain. Both perceived social benefits and negative consequences of concealment were described. Specifically, social motivations to conceal chronic pain symptoms included (1) avoidance of judgment, (2) avoidance of feeling like a burden, and (3) a desire to feel normal. These findings parallel previous evidence of social motivations to conceal disease status observed in other pediatric populations (Sunil George and Lambert, 2015; Galano et al., 2017).

Our sample of adolescents with chronic pain discussed ways that the concealment of their pain symptoms from others was socially beneficial to them. The most endorsed reason was the avoidance of judgment by others, which included medical providers, school personnel, peers and family members. Thus, a primary motivation to hide chronic pain symptoms may be avoiding felt stigma experiences. In particular, the use of concealment as a coping strategy was perceived to be helpful in avoiding the experience of social rejection or exclusion by others, which is consistent with previous research on social relationships and youth with chronic pain (Forgeron et al., 2013). Concealment was also used to avoid a lack of understanding from peers, which has been noted to be a social difficulty for youth with chronic pain (Stinson et al., 2014). Prior research has also documented the fear of social rejection in the context of other stigmatized chronic health populations (Kaushansky et al., 2017), and peer victimization has also been documented in adolescents with chronic pain (Forgeron et al., 2010; Fales et al., 2016). Thus, our findings indicate the need for additional research to better understand pain-related stigma, concealment, and peer relationships among adolescents with chronic pain.

There was also a sense that adolescents with chronic pain felt that their physical and emotional needs were considered a burden to others. The experience of feeling like a burden has been identified as one aspect of internalized stigma (Vervoort et al., 2014). In our sample, adolescents described their perceptions that others within their social environment would not have the capacity to handle their needs or lose interest in their friendship over time due to their chronic pain. This notion was a motivating factor in their concealment of chronic pain symptoms, and suggests the importance of future work to better understand the ways in which adolescents with chronic pain may internalize stigma. Moreover, adolescents with chronic pain were motivated to conceal their pain symptoms out of a desire to be treated normally, which has emerged as a theme in other qualitative research in youth with chronic pain (Meldrum et al., 2009). If peers were not aware of the pain that the adolescent was experiencing, the adolescent could experience feeling “normal.” Future psychological interventions should target communication and advocacy regarding the chronic pain needs to increase support from others.

Whereas adolescents in our sample identified several beneficial aspects of concealment as a coping strategy, there were several descriptions that indicated that concealment also may have harmful consequences; specifically, social isolation and increased cognitive burden. Social relationships for adolescents with chronic pain can be challenging, and it appears that concealment of pain symptoms may allow for some feelings of normalcy and social support. Social support is a protective factor in the management of chronic health conditions in young populations. However, adolescents with chronic pain who conceal their conditions may inadvertently create further social isolation for themselves by silently suffering in their pain alone. This social isolation has negative health implications for youth with chronic pain (Steele et al., 2002; Forgeron et al., 2010). Clinical interventions should focus on improving social connectedness between adolescents with chronic pain and their primary support groups, specifically friends and family members, in order to reduce social isolation and consequential negative health outcomes.

Another negative consequence of concealment described by our sample was an increase in cognitive burden needed to manage concealment. Cognitive burden in the context of stigma concealment has also been mentioned among individuals of other stigma identities (Quinn, 2018). Adolescents with chronic pain need to stay vigilant to social cues that they may be devalued due to their pain by others, as seen in other stigmatized conditions (Chaudoir and Quinn, 2010). This vigilance can contribute to cognitive and emotional fatigue as the adolescent strains to internalize their pain experience and attempt to behave as if not in pain. The findings of this study revealed the possibility of this process, but more research is needed to determine the presence and frequency of this cognitive vigilance, and its impact on daily and school functioning in adolescents with chronic pain, particularly their psychological wellbeing, concentration and school performance. Additional research is also needed to assess the cognitive burden of concealment in the context of intersecting pain-related stigma and other stigmatized identities (Benson et al., 2017), such as youth who identify as a sexual or gender minority.

Finally, a potential negative implication of concealment that was not discussed by participants in our study, is the potential for medical neglect. If adolescents are concealing their pain episodes, they may fail to seek or receive appropriate or adequate medical care when needed. It may be that social consequences (e.g., peer rejection) of disclosing their pain symptoms are more salient to adolescents than potential medical consequences, especially given their developmental stage and the importance of social relationships and peer acceptance during this time period. Nevertheless, it seems warranted for future research to examine links between concealment of chronic pain and medical consequences/quality of care in adolescents.

Several limitations of this study should be noted. Our sample consisted of mostly female adolescents and only one male and one gender fluid participants, which limits our ability to describe stigma concealment perspectives across gender identifications. Future research should prioritize samples with increased gender and racial/ethnic diversity to determine how experiences of pain-related stigma differ for adolescents across diverse backgrounds. We also did not capture more specific information about the frequency and intensity of their pain condition, which may have provided insight into how concealment may be experienced differently based on disease characteristics. The analysis of the current study is also limited by the exploration of codes that were extracted from the larger study. It is possible that more directed questions about concealment may increase the depth of knowledge on this coping strategy. A limitation for inductive content analysis is that the analysis can be subject to coder bias, which was managed by continual reference to the data. Due to the cross-sectional nature of the study, we could describe the experience of adolescents with chronic pain in the context of pain-related stigma and concealment, but could not directly link this coping strategy to health outcomes. We also could not include the experience of adolescents who suffer with chronic pain who have not received a formal diagnosis from a pain clinic, for which concealment from pain-related stigma may be intensified.

While our qualitative study suggests the presence of, and motivations for, concealment among adolescents with chronic pain in the context of pain-related stigma, future research should examine in what context or relationships adolescents with chronic pain may feel comfortable disclosing their health status or symptoms. The decision to seek support from others has been studied in other populations with concealable stigmas (Barned et al., 2016; Roberts et al., 2020) and pediatric chronic conditions (Barned et al., 2016). The decision to disclose health status can vary based on situational contexts, which creates challenges in the measurement of stigma concealment due to its existence along a continuum (Pihlaskari et al., 2020). Further, it is important to note that communicating about disclosure of chronic medical conditions can be challenging even with less stigmatizing conditions (Roberts et al., 2020), and the role of communication difficulties related to one’s health status may be a contributing factor in lack of disclosure (Remedios and Snyder, 2018; Puhl et al., 2019). A greater understanding of these communication barriers in adolescents with chronic pain may lead to appropriate interventions to improve communications and outcomes.

## Data Availability Statement

The datasets presented in this article are not readily available because the transcripts are not available publicly due the need to keep the participants anonymous and protect health information. Requests to access the datasets should be directed to EW, ewakefield@connecticutchildrens.org.

## Ethics Statement

The studies involving human participants were reviewed and approved by the Connecticut Children’s IRB. Written informed consent to participate in this study was provided by the participants’ legal guardian/next of kin.

## Author Contributions

EW, ML, RP, and WZ developed the purpose and design of the study. EW contributed to the implementation of the study, conducted the analyses and wrote the manuscript with input from all authors. All authors contributed to the article and approved the submitted version.

## Conflict of Interest

The authors declare that the research was conducted in the absence of any commercial or financial relationships that could be construed as a potential conflict of interest.

## References

[B1] Barned C. Stinzi A. Mack D. O’Doherty K. C. (2016). To tell or not to tell: a qualitative interview study on disclosure decisions among children with inflammatory bowel disease. *Soc. Sci. Med.* 162 115–123. 10.1016/j.socscimed.2016.06.023 27344353

[B2] Benson A. Lambert V. Gallagher P. Shahwan A. Austin J. K. (2017). Parent perspectives of the challenging aspects of disclosing a child’s epilepsy diagnosis to others: why don’t they tell? *Chronic Illn.* 13 28–48. 10.1177/1742395316648749 27170783

[B3] Betsch T. A. Gorodzinsky A. Y. Finley G. A. Sangster M. Chorney J. (2017). What’s in a name? health care providers’ perceptions of pediatric pain patients based on diagnostic labels. *Clin. J. Pain* 33 694–698. 10.1097/AJP.0000000000000454 27922842

[B4] Camacho G. Reinka M. A. Quinn D. M. (2020). Disclosure and concealment of stigmatized identities. *Curr. Opin. Psychol.* 31 28–32. 10.1016/j.copsyc.2019.07.031 31430614

[B5] Chaudoir S. R. Quinn D. M. (2010). Revealing concealable stigmatized identities: the impact of disclosure motivations and positive first-disclosure experiences on fear of disclosure and well-being. *J. Soc. Issues* 66 570–584. 10.1111/j.1540-4560.2010.01663.x 26160985 PMC4494783

[B6] Fales J. Rice S. Palermo T. (2016). (231) daily peer victimization experiences predict functional disability among adolescents seeking treatment for chronic pain: a prospective diary study. *J. Pain* 17:S33. 10.1016/J.JPAIN.2016.01.135

[B7] Forgeron P. A. Evans J. McGrath P. J. Stevens B. Finley G. A. (2013). Living with difference: exploring the social self of adolescents with chronic pain. *Pain Res. Manag.* 18:e115–e123. 10.1155/2013/120632 24308027 PMC3917802

[B8] Forgeron P. A. King S. Stinson J. N. McGrath P. J. MacDonald A. J. Chambers C. T. (2010). Social functioning and peer relationships in children and adolescents with chronic pain: a systematic review. *Pain Res. Manag.* 15 27–41. 10.1155/2010/820407 20195556 PMC2855293

[B9] Frable D. E. S. Platt L. Hoey S. (1998). Concealable stigmas and positive self-perceptions: feeling better around similar others. *J. Pers. Soc. Psychol.* 74 909–922. 10.1037/0022-3514.74.4.909 9569651

[B10] Galano E. Turato E. R. Succi R. C. de Souza Marques H. H. della Negra M. da Silva M. H. (2017). Costs and benefits of secrecy: the dilemma experienced by adolescents seropositive for HIV. *AIDS Care* 29 394–398. 10.1080/09540121.2016.1248891 27802773

[B11] Goffman E. (1963). *Stigma: Notes on the Management of Spoiled Identity.* New York, NY: Simon and Schuster.

[B12] Hsieh H. F. Shannon S. E. (2005). Three approaches to qualitative content analysis. *Qual. Health Res.* 15 1277–1288. 10.1177/1049732305276687 16204405

[B13] Igler E. Lang A. Balistreri K. Sejkora E. Drendel A. Davies W. H. (2020). Parents reliably identify pain dismissal by pediatric providers. *Clin. J. Pain* 36 80–87. 10.1097/AJP.0000000000000776 31764165

[B14] Kaushansky D. Cox J. Dodson C. McNeeley M. Kumar S. Iverson E. (2017). Living a secret: disclosure among adolescents and young adults with chronic illnesses. *Chronic Illn.* 13 49–61. 10.1177/1742395316655855 27343016

[B15] Krueger R. A. Casey M. (2015). *Focus Groups: A Practical Guide for Applied Research*, 5th Edn. Thousand Oaks, CA: SAGE Publications Inc.

[B16] King S. Chambers C. T. Huguet A. MacNevin R. C. McGrath P. J. Parker L. (2011). The epidemiology of chronic pain in children and adolescents revisited: a systematic review. *Pain* 152 2729–2738. 10.1016/j.pain.2011.07.016 22078064

[B17] Laird K. T. Smith C. A. Hollon S. D. Walker L. S. (2020). Validation of the health-related felt stigma and concealment questionnaire. *J. Pediatr. Psychol.* 45 509–520. 10.1093/jpepsy/jsaa030 32388554 PMC7234182

[B18] Major B. Dovidio J. F. Link B. G. Calabrese S. K. (2018). “Stigma and its implications for health: introduction and overview,” in *The Oxford Handbook of Stigma, Discrimination, and Health*, eds Major B. Dovidio J. F. Link B. G. (New York, NY: Oxford University Press), 3–28. 10.2174/9781681082233116010003

[B19] Meldrum M. L. Tsao J. C.-I. Zeltzer L. K. (2009). “I can’t be what i want to be”: children’s narratives of chronic pain experiences and treatment outcomes. *Pain Med.* 10 1018–1034. 10.1111/j.1526-4637.2009.00650.x 19594848 PMC2758095

[B20] Morgan D. Krueger R. (1993). “When to use focus groups and why,” in *Successful Focus Groups: Advancing the State of the Art*, ed. Morgan D. L. (London: SAGE Publications Inc), 3–19. 10.4135/9781483349008

[B21] Neville A. Jordan A. Beveridge J. K. Pincus T. Noel M. (2019). Diagnostic uncertainty in youth with chronic pain and their parents. *J. Pain* 20 1080–1090. 10.1016/j.jpain.2019.03.004 30904516

[B22] Neville A. Noel M. Clinch J. Pincus T. Jordan A. (2020). Drawing a line in the sand’: physician diagnostic uncertainty in paediatric chronic pain. *Eur. J. Pain* 25 430–441. 10.1002/ejp.1682 33064862

[B23] Pennebaker J. W. Beall S. K. (1986). Confronting a traumatic event: toward an understanding of inhibition and disease. *J. Abnorm. Psychol.* 95 274–281. 10.1037/0021-843x.95.3.274 3745650

[B24] Pihlaskari A. K. Anderson B. J. Eshtehardi S. S. McKinney B. M. Marrero D. G. Thompson D. (2020). Diabetes disclosure strategies in adolescents and young adult with type 1 diabetes. *Patient Educ. Couns.* 103 208–213. 10.1016/j.pec.2019.08.019 31447195 PMC12168200

[B25] Puhl R. M. Himmelstein M. S. Watson R. J. (2019). Weight-based victimization among sexual and gender minority adolescents: findings from a diverse national sample. *Pediatr. Obes.* 14:e12514. 10.1111/ijpo.12514 30729734 PMC7183795

[B26] Quinn D. M. (2018). “When stigma is concealable: the costs and benefits for health,” in *The Oxford Handbook of Stigma, Discrimination, and Health*, eds Major B. Dovidio J. F. Link B. G. (New York, NY: Oxford University Press), 287–300.

[B27] Quinn D. M. Chaudoir S. R. (2009). Living with a concealable stigmatized identity: the impact of anticipated stigma, centrality, salience, and cultural stigma on psychological distress and health. *J. Pers. Soc. Psychol.* 97 634–651. 10.1037/a0015815 19785483 PMC4511710

[B28] Quinn D. M. Earnshaw V. A. (2013). Concealable stigmatized identities and psychological well-being. *Soc. Personal. Psychol. Compass* 7 40–51. 10.1111/spc3.12005 23730326 PMC3664915

[B29] Quinn D. M. Weisz B. M. Lawner E. K. (2017). Impact of active concealment of stigmatized identities on physical and psychological quality of life. *Soc. Sci. Med.* 192 14–17. 10.1016/j.socscimed.2017.09.024 28941787

[B30] Remedios J. D. Snyder S. H. (2018). Intersectional oppression: multiple stigmatized identities and perceptions of invisibility, discrimination, and stereotyping. *J. Soc. Issues* 74 265–281. 10.1111/josi.12268

[B31] Roberts C. M. Gamwell K. L. Baudino M. N. Grunow J. E. Jacobs N. J. Tung J. (2020). The contributions of illness stigma, health communication difficulties, and thwarted belongingness to depressive symptoms in youth with inflammatory bowel disease. *J. Pediatr. Psychol.* 45 81–90. 10.1093/jpepsy/jsz084 31633787

[B32] Ross A. C. Simons L. E. Feinstein A. B. Yoon I. A. Bhandari R. P. (2018). Social risk and resilience factors in adolescent chronic pain: examining the role of parents and peers. *J. Pediatr. Psychol.* 43 303–313. 10.1093/jpepsy/jsx118 29048554

[B33] Steele C. M. Spencer S. J. Aronson J. (2002). Contending with group image: the psychology of stereotype and social identity threat. *Adv. Exp. Soc. Psychol.* 34 379–440. 10.1016/s0065-2601(02)80009-0

[B34] Stinson J. N. Lalloo C. Harris L. Isaac L. Campbell F. Brown S. (2014). ICanCope with Pain^TM^: user-centred design of a web- and mobile-based self-management program for youth with chronic pain based on identified health care needs. *Pain Res. Manag.* 19 257–265. 10.1155/2014/935278 25000507 PMC4197753

[B35] Sunil George M. Lambert H. (2015). I am doing fine only because i have not told anyone: the necessity of concealment in the lives of people living with HIV in india. *Cult. Health Sex.* 17 933–946. 10.1080/13691058.2015.1009947 25706959 PMC4772686

[B36] Tanna V. Heathcote L. C. Heirich M. S. Rush G. Neville A. Noel M. (2020). Something else going on? diagnostic uncertainty in children with chronic pain and their parents. *Children* 7:165. 10.3390/children7100165 33020423 PMC7599582

[B37] Uysal A. Lu Q. (2011). Is self-concealment associated with acute and chronic pain? *Health Psychol.* 30 606–614. 10.1037/a0024287 21728422

[B38] Vervoort T. Logan D. E. Goubert L. de Clercq B. Hublet A. (2014). Severity of pediatric pain in relation to school-related functioning and teacher support: an epidemiological study among school-aged children and adolescents. *Pain* 155 1118–1127. 10.1016/j.pain.2014.02.021 24631587

[B39] Wakefield E. O. Zempsky W. T. Puhl R. M. Litt M. D. (2018). Conceptualizing pain-related stigma in adolescent chronic pain: a literature review and preliminary focus group findings. *Pain Rep.* 3:e679. 10.1097/PR9.0000000000000679 30324171 PMC6172824

[B40] Wegner D. M. Lane J. D. (1995). “From secrecy to psychopathology,” in *Emotion, Disclosure, & Health*, ed. Pennebaker J. W. (Washington, DC: American Psychological Association), 25–46. 10.1037/10182-002

